# Acupuncture Treatment in Elderly People with Sarcopenia: Effects on the Strength and Inflammatory Mediators

**DOI:** 10.1155/2019/8483576

**Published:** 2019-01-27

**Authors:** Gisele Soares Mendes Damasceno, Thaís Helena Machado Marçal Teixeira, Vinicius Carolino de Souza, Tiago Sousa Neiva, Karina Prudente Pereira, Maria de Fátima Teles Landim, Gislane Ferreira de Melo, Juliana de Faria Fracon e Romão, Otávio Tolêdo Nóbrega, Gustavo de Azevedo Carvalho

**Affiliations:** ^1^Universidade Católica de Brasília, Brasília, Brazil; ^2^Fundação de Ensino e Pesquisa em Ciências da Saúde, Brasília, Brazil; ^3^Universidade de Brasília, Brasília, Brazil; ^4^Secretaria de Estado de Saúde do Distrito Federal, Brasília, Brazil

## Abstract

**Introduction:**

Sarcopenia is defined as the progressive loss of skeletal muscle mass, associated with aging. A multidisciplinary approach has been increasingly prioritized in elderly care. A technique that has been widely used by the seniors is acupuncture.

**Objectives:**

To analyse the effects of acupuncture in muscle strength and in inflammatory markers of older people with sarcopenia.

**Methods:**

The sample was composed by 53 elderly people, aged over 60 years. Inclusion criteria were as follows: male and female seniors, sedentary and who were not under acupuncture treatment during the survey period. Assessment of body composition, handgrip strength, and functional test and IL-6, IL-10, and TNF-*α* cytokines analyses were performed. After verification of the physical examination, the subjects were divided into two groups (sarcopenic and nonsarcopenic). The first group was then randomized (by drawing lot) to be further divided into two subgroups: G1, composed of sarcopenic elderly people who received acupuncture intervention, and G2, composed of sarcopenic elderly people who did not receive intervention. The nonsarcopenic elderly people composed the group 3 (G3) and did not receive acupuncture intervention. ANOVA Split Plot was performed for intergroup comparison. For intragroup evaluation, ANOVA was conducted for repeated measures. For the delta values, ANCOVA was performed with the pretest as covariant. A *p* < 0.05 significance level was adopted.

**Results:**

26 older people concluded the collections. There was no statistically significant difference between the G1 group and the other ones regarding the assessed variables (muscle mass, muscle strength, functionality, and inflammatory markers).

**Conclusion:**

The results allow us to infer that it is possible that the conducted intervention protocol has not produced any significant effects in the studied population. UTN number: RBR-8df2h4.

## 1. Introduction

Sarcopenia is defined as the progressive loss of skeletal muscle mass, associated with aging, with reduction of the number and size of muscle fibers, and with parallel decrease in muscular strength and endurance [1–3]. It may lead to falls, decreased functional-physical capacity, and increased energy expenditure, causing serious impacts on the health of the elderly.

With aging, the muscle regeneration process becomes deficient, as the production of endogenous stem cells becomes ineffective, and functional muscle replacement by adipose and fibrous tissue occurs. Moreover, there is a reduction in the capacity of muscle reinnervation and loss of alpha motor neurons of the spinal cord, causing degeneration of axons and reduction in the motor unit recruitment [4].

Sarcopenia establishes its symptoms especially in physically inactive individuals, but it is also seen in individuals who remain physically active throughout their lives, causing a diversity of functional alterations found in these elderly persons. A sedentary lifestyle plays an important role in the functional losses associated with aging. Individuals who do not exercise for most of their lives present bigger risks of having a deficit in joint mobility, in maintenance or increase in muscle mass, and in static and dynamic balance in old age [5].

For the treatment of sarcopenia, it is recommended the practice of physical exercises, particularly those for muscle strengthening [2, 5, 6], associated with dietary intervention, with protein intake [3, 7–9]. When necessary, pharmacological intervention is made via hormone replacement therapy, with testosterone, estrogen, and growth hormone [10].

Increasingly, the multidisciplinary care approach for the elderly has been prioritized due to their diverse demands and needed care to maintain a good quality of life. A technique that has been widely used by the seniors is acupuncture [11]. This millenary practice of Oriental medicine can help treat various diseases. In addition to being recognized and encouraged by the World Health Organization as a supplementary therapy, it consists of a low-cost technique, with no significant side effects and with proven efficacy in treating several clinical conditions [12].

In a study in which the maximum dynamic and explosive strength, anaerobic endurance, and speed in young high-performance sprinters before and after acupuncture were analysed, significant and clinical improvements in the maximum dynamic strength and in the explosive strength were observed, with the conclusion that acupuncture improved the physical performance of the studied population [13].

Furthermore, Chae et al. investigated the immunomodulatory effects of acupuncture through its action on circulating levels of different cytokines in mice and they discovered that acupuncture can cause significant decrease in serum levels of interleukin 6 (IL-6) and tumor necrosis factor alpha (TNF-*α*). It is necessary to investigate whether these changes can also be found in elderly people and to deepen knowledge in this area, with the aim of reducing the deleterious effects of aging, such as sarcopenia and immunosenescence [14].

Considering the impacts caused by sarcopenia on the functional-physical capacity of elderly people and the need for alternatives that reduce organic and functional losses, the objective of this study was to analyze the effects of acupuncture on muscle strength and on inflammatory markers of elderly people with sarcopenia.

## 2. Materials and Methods

### 2.1. Subjects

A quantitative experimental blind longitudinal study was conducted. The sample was initially composed by 53 elderly people, aged over 60 years. In a previous survey conducted in the “Granja do Torto, Distrito Federal,” 149 seniors were found in this community. The recruitment of participants was carried out by telephone contact with the aforementioned elderly people. In this contact, they were invited to participate in the study and in a lecture, scheduled in advance, that was offered to every invited person, with the objective of explaining the study.

Inclusion criteria were as follows: male and female seniors, sedentary and who were not under acupuncture treatment during the survey period. Individuals who were afraid of needles, with mobility problems that prevented them to attend the data collection and processing location, and who presented difficulties of understanding the treatment and the applied tests were excluded from this study. Subjects who presented diseases that affect muscle mass and strength, such as sequelae from cerebral vascular accident, multiple sclerosis, muscular dystrophy, Parkinson's disease, among others, were also excluded.

This project was approved by the Research Ethics Committee of the Universidade Católica de Brasilia. The elderly people who voluntarily accepted to participate in the project signed the freely given informed consent form and were told to return in a scheduled time of a specific day for the performance of the procedures that are described below.

### 2.2. Physical Examination: Muscle Mass, Muscle Strength, and Functionality

All data collection procedures were performed in the Community Hall of Granja do Torto, Distrito Federal, in the morning, in an illuminated and ventilated office booked for that evaluation. Initially, the level of physical activity was evaluated via the short version of the International Physical Activity Questionnaire, IPAQ, validated for the Brazilian population [15]. Only seniors classified as sedentary or irregularly active were selected. All participants were told not to exercise during the survey period. Thereafter, anthropometric data were collected, consisting of an assessment of body mass (digital scale, Filizola®) and of height (wall stadiometer, CardioMed®), with the assessment of the body composition of each participant, performed by a tetrapolar bioimpedance test (Biodynamics® 310). Each participant was previously instructed not to eat big meals up to four hours before the examination, to avoid caffeine and alcohol consumption in the 24 hours before it, and to wear light clothing during the procedure.

For analysis of functional capacity, the TUG test was performed. Before the timed test, the patient executed it once as training. The performance was assessed in seconds. The timing started after the word “go,” while the individual was still seated and ended as soon as the subject sat down again. Physical assistance was not allowed during the test [16].

Then the handgrip strength was measured using a calibrated dynamometer (Jamar®). The dynamometer was adjusted in the second position, considered to be more efficient for strength tests. Three alternated measures between the limbs were conducted, with one-minute rest between measurings. The values of the three measurings were recorded, and the highest one was considered [17, 18].

All data collection procedures mentioned before were performed by a sole evaluator, physiotherapist with more than five years of experience and trained to apply those tests, unlike the principal investigator during all the survey, who was not aware of which group each participant was part of, what characterized the study as a blind one.

### 2.3. Laboratory Tests: Inflammatory Mediators

Serum samples of each individual were collected and processed for the analysis of inflammatory mediators by a trained laboratory technician, blinded to the physical examination. The samples were stored in a freezer at −80°C for later dosage and analysis of IL6, IL10, and TNF-*α* cytokines by means of an immunoenzymatic assay (enzyme-linked immunosorbent assay), specific to each mediator. The dosages were held in duplicate, using a commercial kit (BioLengend®). Data were analysed in the Immunogerontology Laboratory of the Universidade Católica de Brasília (UCB).

### 2.4. Intervention

After verification of the physical examination, the subjects were divided into two groups (sarcopenic and nonsarcopenic), according to the recommendations of the European Working Group on Sarcopenia in Older People (EWGSOP). This group suggests as “sarcopenic” individuals who present decrease of at least two out of three assessed parameters: muscle mass, muscle strength, and functionality. The cuoff points used in this study were as follows [19]:

  BIA: men: 8.87 kg/m^2^; women: 6.42 kg/m^2^
  Handgrip: men: <30 kg; women: <20 kg
  TUG: >0.8 M/S

The first group was then randomized (by drawing lot) to be further divided into two subgroups: G1, composed of sarcopenic elderly people who received acupuncture intervention, and G2, composed of sarcopenic elderly people who did not receive intervention. The nonsarcopenic elderly people composed group 3 (G3) and did not receive acupuncture intervention (data from this group will be used as a normal development parameter for the sample analysed in this study). All the subjects were contacted again to be informed of their situation and of time and place for performance of the next procedures.

G1 had 24 acupuncture sessions. Sessions were held three times a week, in-between days, in the morning, conducted by an acupuncturist physiotherapist with more than five years of experience. Disposable sterile needles with the size of 25 × 30 mm (Han sol) were used. Treatment was performed employing tonification acupoints, according to the recommendations of Luna and Fernandes Filho [13] and Maciócia and Ming [20]. The following points were used in tonification: R3, BP3, BP6, VB34, F8, E36, and TA6.

All the seven points were bilaterally used. The De Qi sensation (needle sensation) was obtained in all sessions. The participant remained lying with the needles in the points for 20 minutes.

New bioimpendance, handgrip, and TUG tests, as well as blood collection, were performed after 12 sessions, 24 sessions, and 30 days after the end of the intervention, totaling four collections throughout the survey. G2 and G3 received no intervention and were informed by telephone of the dates for data collection. Individuals who have missed two or more test days were excluded from the study.

### 2.5. Statistical Analysis

Collected data were inputted in an Excel 2010 (for Windows) spreadsheet and processed in the Statistical Package for the Social Sciences (SPSS) 20.0 software. ANOVA Split Plot was performed for intergroup comparison. For intragroup evaluation, ANOVA was conducted for repeated measures. For the delta values, ANCOVA was performed with the pretest as covariant. A *p* < 0.05 significance level was adopted.

## 3. Results

The study began with 53 participants; however, only 26 concluded the collection, due to sample loss. G1 (*n*=11) presented an average age of 72 ± 7.9 years, lean mass weight of 38.9 ± 6.7 kg, and body fat percentage of 33.4 ± 6.1%. G2 (*n*=4) presented an average age of 63.5 ± 3.3 years, lean mass weight of 38.1 ± 6.3 kg, and body fat percentage of 40.0 ± 7.0%. G3 (*n*=12) presented an average age of 67.4 ± 7.7, lean mass weight of 49.5 ± 8.7 kg, and body fat percentage of 36.4 ± 6.9%. The groups showed normal distribution.

**Figure 1:**
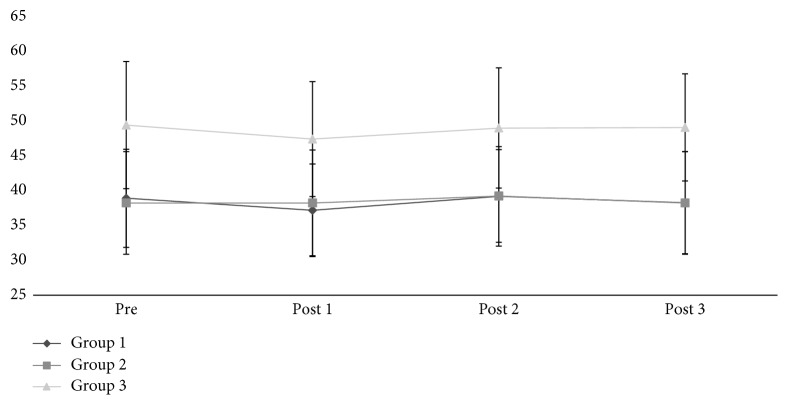
Lean body mass weight—groups G1, G2, and G3.

In the evaluation of lean body mass (Figure 1), G1 and G2 presented significantly lower values than G3 in all evaluated moments (*p*=0.002). The groups presented no significant changes during the study period, with levels of lean body mass remaining stable (*p*=0.348). G1 presented similar behavior to the other groups who received no intervention.

**Figure 2:**
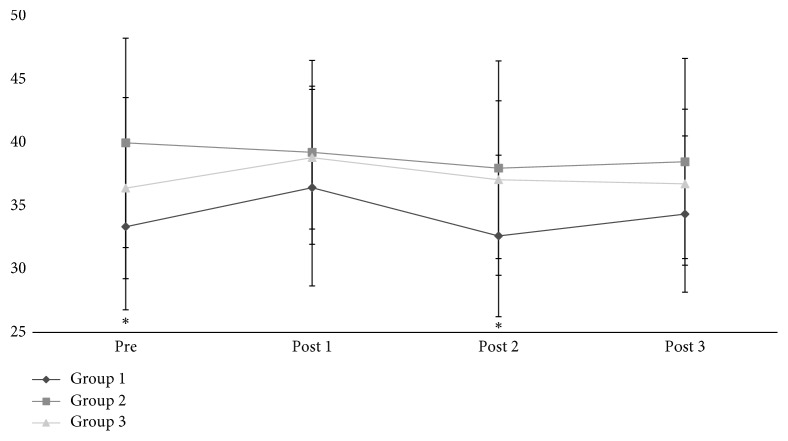
Body fat percentage—groups G1, G2, and G3. ^*∗*^Significant statistical difference between G1 and G2.

Figure 2 shows the body fat percentage. Even though the values of the three groups are above the recommended levels, G1 had the lowest values, when compared to the other groups, with significant difference in comparison to G2 (*p*=0.027) at Pre and Post 1 moments. However, the acupuncture intervention was not capable of promoting important changes in the body fat levels of G1 (*p*=0.358).

Handgrip strength values can be seen in Table 1. G2 had lower values than the other groups, with significant difference at Post 3 moment in comparison to G3 (*p*=0.002). In spite of no statistical difference, G2 and G3 presented a downward trend in the handgrip strength, while G1 presented more stable values in both limbs.

**Figure 3:**
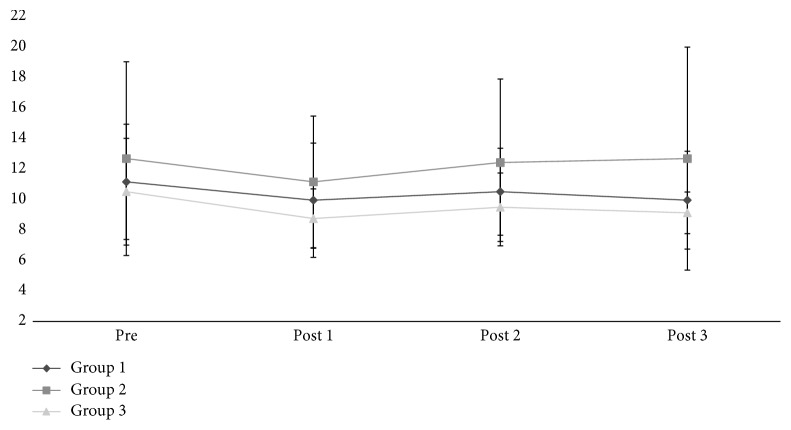
TUG test time—G1, G2, and G3.

In the functionality assessment (Figure 3), G2 presented a slower TUG test time, but with no statistical significance. This group showed an upward trend in time, while G1 and G3 presented similar behavior, with nonsignificant test time reduction (*p*=0.86). From the obtained values, the delta was calculated, with Pre moment as covariant, and there was no significant difference.

With respect to IL-6, all groups showed similar behavior and a statistical difference at Post 2 moment in comparison to Pre moment noted (*p*=0.05). Even with a slight drop in the values, they were still higher than expected (2.08 pg/ml). Data can be seen in Table 2.

IL-6 and TNF-*α* values usually present direct correlation. In this study, there was no relationship between the behavior of IL-6 and TNF-*α*. The groups presented no statistical difference between them or between the evaluated moments. All groups presented lower values than ideal (4.12 pg/ml).

IL-10 values showed a significant drop in G2 at Post 3 moment in comparison to the baseline value (*p* ≤ 0.001), indicating loss of anti-inflammatory cytokines that provide protection against the oxidative effects of aging. G1 and G3 presented a less-significant decrease of IL-10 during the evaluated period. All groups concluded the study with lower values than ideal (3.5 pg/ml). From the obtained values, the delta was calculated, with Pre moment as covariant, and there was no significant difference.

## 4. Discussion

This study evaluated the effects of acupuncture in elderly patients with sarcopenia. At the end of the treatment, the protocol was not capable of promoting significant changes in the group that received intervention in comparison to the baseline values or in comparison to the control groups in any assessed variable. In the literature review, it was not possible to detect any similar studies that employed acupuncture in elderly people with the objective of gaining muscle mass or strength. The studies that have this purpose usually are conducted with athletes or young individuals, showing positive results [21, 22].

Another study evaluated the effects of using acupuncture in local muscle endurance of upper limbs in weight training practitioners. The sample was composed by 40 individuals randomly divided into the Intervention Group and the Control Group. Arm flexion test was conducted in order to quantify the local muscle endurance of the upper limbs in the participants, and acupuncture was applied at specific points with the objective of promoting a possible improvement in their performance. At the end of the study, the individuals that were subjected to acupuncture had an increase in their performance, in comparison to the Control Group [23].

It is possible that acupuncture bolsters physiological processes that are already benefited by physical exercises, as in the case of active individuals; however, in the case of individuals who already present an existing muscle loss and who do not practice any physical activity, such as the elderly people evaluated in this study, perhaps this mechanism is not possible, or it requires a longer time to present results.

Acupuncture meridians pass through the body nourishing and modulating muscle activity. Magnetic resonance studies have shown that stimulation of acupuncture points stimulates or reduces activity in the brain areas corresponding to the motor command, activating type A fibers immediately after needle stimulation.

No studies evaluating acupuncture stimulation for muscle mass gain in the elderly were found. However, considering the function of the points used in the present study, it is suggested that the gain of muscle strength occurs due to increased blood irrigation, the promotion of elasticity of muscle fibers, and improvement of muscular contractility. More studies are needed to study the muscle function stimulated by acupuncture in the elderly.

In a study that analysed the effects of a workout program in walking and functional mobility of seniors, the participants did circuit training exercises for six months with the objective of improving body schema, muscle strength, balance, and walking. Walking speed and functional mobility were evaluated before and after the intervention. The workout program improved the functional performance of elderly people and positively modified the walking variables [24].

This study did not present results that are similar to the ones observed in studies with resisted exercises. Even though acupuncture is a technique that aims at promoting the energy rebalancing to the individual's health establishment, it is a supplementary measure and it should not replace other indicated treatments [11]. Probably acupuncture treatment associated with other interventions, such as physical activities and a balanced diet, deliver different results to the studied population.

There is no consensus regarding the points used for strength and muscle mass gain [23]. In this study, the choice of acupoints was based on the literature, energetic function, indications, and location. A smaller number of points were used to avoid conflicts with the energetic function. The duration of sessions was 20 minutes, with the objective of tonifying the points.

The used acupoints are in accordance with the objective of the study, since in the framework of sarcopenia, besides Kidney Qi (energy) Deficiency (usual in aging), Spleen-Pancreas Yang deficiency also occurs. All the points were used aiming to tone one or more affected organs.

IL-6 values observed in this study are noteworthy. Both groups of elderly people (the sarcopenic and the nonsarcopenic ones) presented high IL-6 values. Elevated IL-6 values are strongly associated with increased mortality rate in elderly people [25]. The adipocytes and macrophages are responsible for some of the IL-6 production, although the mechanisms of IL-6 induction by excess of adipose tissue are still unknown [26].

This is a possible cause for the results found, as the body fat percentage in the three evaluated groups were higher than ideal. There was no significant difference in the fat percentage between the evaluated groups; however, it is important to emphasize that despite the fact that the nonsarcopenic elderly people had an elevated fat percentage, their lean mass was also in a higher amount.

Even though IL-6 and TNF-*α* values are positively correlated in other studies [27–29], in this study, there was no correlation because despite the elevated IL-6 values in all the evaluated groups throughout the survey, TNF-*α* registered normal values in all the groups.

As to the anti-inflammatory IL-10 cytokine, there was no difference between the evaluated groups, despite the fact that G3 had levels that were higher and within the expected values. Balance between circulating concentration of pro- and anti-inflammatory cytokines is considered an important means of controlling chronic inflammation, being subject to regulation by genetic factors and lifestyle [30]. Considering such information, it is possible that sarcopenic elderly people of this study are at high risk of developing other diseases, since the levels of pro- and anti-inflammatory cytokines are not in balance.

The stimulation with acupuncture modulates several inflammatory markers both by suppressing its receptors and by acting on the cholinergic anti-inflammatory pathway that involves the expression of catecholamines. No studies were found to evaluate the modulation of inflammatory markers in the elderly after stimulation with acupuncture. It is suggested that this modulation occurs due to a homeostatic balance promoted by acupuncture, by reducing the synthesis of proinflammatory factors, and by increasing the production of anti-inflammatory factors. Further studies are needed to explore acupuncture activity in inflammatory markers of elderly patients with sarcopenia.

Although it was not the objective of this study, it was observed that the elderly people who were subjected to acupuncture treatment reported subjective improvement, namely, eight volunteers reported decreased joint and muscle pains, and two elderly patients who needed crutches or cane to walk discontinued the use of the devices in the first ten sessions. Six participants reported improved performance of daily tasks, and all the members of G1 reported sense of well-being with acupuncture, even after 30 days of treatment interruption. These reports demonstrate that even though the results are not statistically significant, acupuncture was capable of promoting positive changes and improvement in the quality of life of the evaluated elderly people. Future studies may assess whether a longer treatment in a larger sample is capable of promoting significant results.

This study showed some limitations. The sample size and sample loss during the survey may have interfered in the obtained results. At the end, the groups contained a very small sample, especially group 2, with only four participants, all female. This can severely affect the results, as women have less muscle mass, higher fat percentage, and smaller predictive values for the evaluated tests, when compared to men.

Besides, considering that sarcopenia is a chronic condition that is established over the years, it is possible that the intervention period defined in this study may have been insufficient. The clinical practice allows us to observe that chronic diseases require more time of acupuncture treatment to present effective results.

The standard treatment for sarcopenia advocates strengthening exercises and dietary interventions. Studies have shown that resisted exercises programs for frail elderly people start showing significant results, such as increased muscle strength and decreased risk of falling, between 8 and 24 weeks of treatment, conducted twice to three times a week [30–33]. Studies that evaluated the nutritional changes with or without protein supplementation in the diet of sarcopenic elderly people observed the behavior of muscle mass between 10 and 18 weeks of intervention [34–36]. These studies reinforce the hypothesis of this survey that the acupuncture treatment requires a longer period to present more significant results.

## 5. Conclusion

This study aimed at evaluating the effects of acupuncture in elderly patients with sarcopenia, and the results allowed us to infer that it is possible that the intervention protocol that was used has not shown significant effects in the evaluated population, despite the remarkable results that were reported by the participants. It is recommended that further studies with a significant sample be conducted, as well as a comparison of the effects of acupuncture with physical activities and interventions on the diet of elderly people.

## Figures and Tables

**Table 1:** Handgrip strength: left upper limb—G1, G2, and G3.

	LUL			RUL		
	G1	G2	G3	G1	G2	G3
Pre	26.00 ± 9.29	23.50 ± 3.11	31.33 ± 10.22	23.18 ± 7.64	23.75 ± 5.19	29.92 ± 8.52
Post 1	25.00 ± 9.44	22.75 ± 2.99	29.50 ± 9.19	21.73 ± 7.84	23.75 ± 4.65	28.42 ± 9.67
Post 2	24.40 ± 8.59	21.50 ± 4.51	28.75 ± 10.31	21.36 ± 6.07	23.75 ± 6.85	27.08 ± 9.71
Post 3	25.40 ± 8.54	20.25 ± 5.32^*∗*^	28.17 ± 9.49	22.00 ± 6.53	21.75 ± 7.04^*∗*^	27.50 ± 10.44

^*∗*^Significant statistical difference between G2 and G3.

**Table 2:** IL-6, TNF-*α*, and IL-10 levels found in G1, G2, and G3: average and standard deviation.

	Pre	Post 1	Post 2	Post 3
IL-6				
G1	3.75 ± 2.17	3.92 ± 3.33	2.31 ± 2.26^†^	3.04 ± 1.76
G2	5.66 ± 5.67	4.95 ± 3.07	2.74 ± 1.79^†^	3.68 ± 0.51
G3	6.43 ± 4.90	5.48 ± 4.09	4.00 ± 2.85^†^	5.16 ± 2.73

TNF-*α*				
G1	0.10 ± 0.20	0.07 ± 0.16	0.04 ± 0.13	0.07 ± 0.13
G2	0.00 ± 0.00	0.00 ± 0.00	0.00 ± 0.00	0.00 ± 0.00
G3	0.03 ± 0.09	0.09 ± 0.31	0.03 ± 0.10	0.02 ± 0.05

IL-10				
G1	2.82 ± 1.05	3.57 ± 0.80	2.63 ± 1.10	2.16 ± 1.20
G2	2.34 ± 0.40	3.14 ± 0.95	2.07 ± 0.83	1.44 ± 0.59^*∗*^
G3	3.74 ± 2.09	3.94 ± 1.58	3.40 ± 1.56	2.43 ± 1.42

^†^Significant difference found in IL-6 in groups at Post 2 moment in comparison to Pre moment. ^*∗*^Statistically significant difference of IL-10 values found in G2 at Post 3 moment in comparison to baseline value.

## Data Availability

The authors declare that these data are exploratory in nature and can be accessed through the description of the data in the article. There are no restrictions on access to these data.
